# COVID‐19 during the index hospital admission confers a ‘double‐hit’ effect on hip fracture patients and is associated with a two‐fold increase in 1‐year mortality risk

**DOI:** 10.1002/msc.1674

**Published:** 2022-08-05

**Authors:** Andrew J. Hall, Nicholas D. Clement, IMPACT Revisited Group, Alasdair M. J. MacLullich, Timothy O. White, Andrew D. Duckworth

**Affiliations:** ^1^ Edinburgh Orthopaedics Royal Infirmary of Edinburgh Edinburgh UK; ^2^ Department of Orthopaedics & Trauma and Usher Institute University of Edinburgh Edinburgh UK; ^3^ Scottish Orthopaedic Research Trust Into Trauma (SORT‐IT) Edinburgh UK; ^4^ Scottish Hip Fracture Audit (SHFA) NHS National Services Scotland Edinburgh UK; ^5^ Department of Orthopaedics Golden Jubilee University National Hospital Clydebank UK; ^6^ Ageing and Health Group Usher Institute University of Edinburgh Edinburgh UK

**Keywords:** audit, COVID‐19, frailty, hip fracture, mortality, nosocomial, orthopaedics

## Abstract

**Purpose:**

The aims were to: (1) determine 1‐year mortality rates for hip fracture patients during the first UK COVID‐19 wave, and (2) assess mortality risk associated with COVID‐19.

**Methods:**

A nationwide multicentre cohort study was conducted of all patients presenting to 17 hospitals in March‐April 2020. Follow‐up data were collected one year after initial hip fracture (‘index’) admission, including: COVID‐19 status, readmissions, mortality, and cause of death.

**Results:**

Data were available for 788/833 (94.6%) patients. One‐year mortality was 242/788 (30.7%), and the prevalence of COVID‐19 within 365 days of admission was 142/788 (18.0%). One‐year mortality was higher for patients with COVID‐19 (46.5% vs. 27.2%; *p* < 0.001), and highest for those COVID‐positive during index admission versus after discharge (54.7% vs. 39.7%; *p* = 0.025). Anytime COVID‐19 was independently associated with 50% increased mortality risk within a year of injury (HR 1.50, *p* = 0.006); adjusted mortality risk doubled (HR 2.03, *p* < 0.001) for patients COVID‐positive during index admission. No independent association was observed between mortality risk and COVID‐19 diagnosed following discharge (HR 1.16, *p* = 0.462). Most deaths (56/66; 84.8%) in COVID‐positive patients occurred within 30 days of COVID‐19 diagnosis (median 11.0 days). Most cases diagnosed following discharge from the admission hospital occurred in downstream hospitals.

**Conclusion:**

Almost half the patients that had COVID‐19 within 365 days of fracture had died within one year of injury versus 27.2% of COVID‐negative patients. Only COVID‐19 diagnosed during the index admission was associated independently with an increased likelihood of death, indicating that infection during this time may represent a ‘double‐hit’ insult, and most COVID‐related deaths occurred within 30 days of diagnosis.

## 1 INTRODUCTION

Patients sustaining a hip fracture are especially vulnerable to contracting and dying from Coronavirus Disease 2019 (COVID‐19) and account for a large number of inpatient deaths from COVID‐19. (Clement, Ng, et al., 2020; Clement, Hall, et al., 2020; Hall et al., 2020) Nevertheless little is known about the prevalence of infection once patients are discharged from the acute hospital stay, and the long‐term effects of COVID‐19 on hip fracture patients remains unclear.

Findings of previous studies by the International Multicentre Project Auditing COVID‐19 in Trauma and Orthopaedics (IMPACT) Group found that, after adjusting for confounding factors, the risk of 30‐day mortality for hip fracture patients with COVID‐19 was three times greater than COVID‐negative patients (COVIDSurg, 2021; Hall, Clement, MacLullich, White, et al., 2021; Hall et al., 2020; Hall & Public Health Scotland, 2021). The multinational IMPACT‐Global Hip Fracture Audit demonstrated that, in the context of COVID‐19 infection, increased age, male sex, frailty, and pre‐existing renal and pulmonary disease were associated with an increased 30‐day mortality risk (Hall, Clement, IMPACT‐Global, et al., 2022; Hall, Clement, MacLullich, Simpson, Johansen, et al., 2022; Hall, Clement, MacLullich, Simpson, White, et al., 2022). However, it is unclear whether the increased risk of death from COVID‐19 is diminished once patients are past the peri‐injury period, whether timing of infection relative to the hip fracture is important, and if the all‐cause mortality for patients affected (at any time) by COVID‐19 is significantly different to that of COVID‐negative patients.

Nosocomial transmission of SARS‐CoV‐2 may have accounted for around half of all cases of COVID‐19 around the time of hip fracture (Hall, Clement, MacLullich, Ojeda‐Thies, et al., 2021; Hall, Clement, MacLullich, White, et al., 2021; Hall, Duckworth, et al., 2021). Despite this, there is little evidence concerning the prevalence of COVID‐19 in patients following discharge from hospital, or the patterns of transmission in this patient group where there is often a need for extended periods of care in inpatient hospital or rehabilitation facilities (‘downstream’ hospitals) or residential care settings.

The primary aim of this nationwide multicentre study was to determine the 1‐year mortality rate for patients with hip fracture during the first UK COVID‐19 wave. Secondary aims were to assess mortality risk associated with: (1) COVID‐19 diagnosed during the initial hip fracture (‘index’) acute hospital admission, and (2) COVID‐19 diagnosed following discharge from the acute hospital.

## 2 METHODS

The International Multicentre Project Auditing COVID‐19 in Trauma and Orthopaedics (IMPACT) Group is a clinical audit network established to facilitate investigation into the effects of the COVID‐19 on hip fracture patients. (Hall, 2020 (2); Hall & Public Health Scotland, 2021) A multicentre observational cohort study was conducted of all patients presenting with a hip fracture to 17 Scottish hospitals between 1^st^ March and 14^th^ April 2020 and data were collected pertaining to patient, injury, and management factors, COVID‐19 status, and outcomes. The findings, which related to risk of acquiring infection, routes of transmission, and 30‐day mortality, were presented in the IMPACT‐Scot Report 2 (Hall, Clement, MacLullich, Ojeda‐Thies, et al., 2021; Hall, Clement, MacLullich, White, et al., 2021; Hall, Duckworth, et al., 2021). The current study collected follow‐up data up to 365 days after the date of index admission (defined as the time between initial presentation and discharge from the acute unit that provided definitive hip fracture care) and included: COVID‐19 status, date of positive COVID‐19 status, discharge destination, readmission to acute hospital, reason for readmission, survival status, primary cause of death, and whether COVID‐19 was a contributing factor to death (defined as death within 28 days of a positive COVID‐19 diagnosis, or COVID‐19 listed on the death certificate) (Office for National Statistics, 2021). Data were collected by clinicians or specialist hip fracture audit coordinators local to each unit in accordance with UK Caldicott principles, and anonymised data were submitted to the central IMPACT Project Lead Team (Caldicott, 1999).

### 2.1 Inclusion and exclusion criteria

The current study included all patients in the IMPACT‐Scot Report 2, which applied the Scottish Hip Fracture Audit of all patients aged 50 years and over and admitted with an acute hip fracture to any of the participating hospitals over the study period (1^st^ March to 15^th^ April 2020). (Hall, Clement, MacLullich, Ojeda‐Thies, et al., 2021; Hall, Clement, MacLullich, White, et al., 2021; Hall, Duckworth, et al., 2021; (SHFA), n.d.) Intracapsular and extracapsular fractures of the proximal femur up to and including the subtrochanteric region (defined as 5 cm distal to the lesser trochanter) were included. Periprosthetic fractures and isolated fractures of the public ramus, acetabulum and greater trochanter were excluded.

### 2.2 Data collection

Follow‐up data were collected from electronic patient records (EPR) by clinicians and specialist audit coordinators in each unit using a bespoke digital IMPACT Revisited data collection tool which utilised data‐validated fields to ensure accuracy and consistency of coding, thus increasing intra‐ and inter‐observer reliability. Data collectors had continuous access to the audit designer (AH) for queries. In addition to the verification of the original IMPACT‐Scot Report 2 dataset, which included demographics, injury factors, comorbidities and laboratory blood results, data were collected for all patients including: length of index acute admission; discharge destination; COVID‐19 status (and date of positive any diagnosis); readmission to acute hospital (and clinical reason); mortality status, and cause of death (including primary cause, whether COVID‐19 was a contributing factor, and whether there was a positive COVID‐19 diagnosis within 28 days of death) (Office for National Statistics, 2021). All variables were examined up to 365 days following the index date of admission.

### 2.3 Statistical methods

Statistical analysis was performed using RStudio (Integrated Development for R. Rstudio, PBC, Boston, MA, USA) using the following packages: finalfit; survival; survminer; table0ne, and tidyverse. Continuous variables were assessed for significant differences between groups using an independent paired *t*‐test, and categorical variables were assessed using a Chi‐square test. Kaplan‐Meier analysis was used to assess 365‐day survival and Log rank was used to compare survival between COVID‐19 negative patients, COVID‐19 positive patients diagnosed during the index stay, and COVID‐19 positive patients diagnosed after discharge from the index stay.

Logistic regression analysis was used to determine factors associated with an increased likelihood of a positive COVID‐19 diagnosis. Factors demonstrating an association (*p* < 0.1) on univariable analysis were included in a multivariable logistic regression model, and factors significantly associated (*p* < 0.05) with an increased likelihood of positive COVID‐19 diagnosis were taken as confounding factors and used to build a proportional hazards model. A Schoenfeld test determined that the proportional hazard assumptions were upheld, and Cox proportional hazard regression analysis was used to assess the independent association of COVID‐19 status and 365‐day mortality. A *p*‐value of <0.05 was defined as statistically significant.

## 3 RESULTS

One‐year follow‐up data were collected from 16 study centres, accounting for 788/833 (94.6%) of patients from the original study, and one study centre did not participate. There were 528 (67.0%) female and 260 (33.0%) male patients with a mean age of 80.5 years (standardised mean difference (SMD) 10.3 years). Table 1 describes the study cohort according to mortality status at 1 year. There were 99/788 (12.6%) patients that died within 30 days of admission for hip fracture, and a total of 242/788 (30.7%) died within 365 days. Cause of death data were available for 155/242 (64.0%) and are described in Table 2. Cause of death was not obtainable in 87/242 (35.0%) patients due to the inaccessibility of the Medical Certificate of Cause of Death.

**TABLE 1:** Patient demographics, nottingham hip fracture score, residence, place of injury, comorbidity, anaesthesiologists (ASA) grade, COVID‐19 status, and time of COVID‐19 diagnosis, according to 365‐day mortality

	Alive		Dead			
Patient factor	(*n* = 546)		(*n* = 242)		*p*‐value^a^	SMD
Mean age, yrs (SD)	79.0	10.4	83.9	9.3	**<0.001**	0.488
Sex, *n* (%)					**<0.001**	0.279
Male	158	28.9	102	42.1		
Female	388	71.1	140	57.9		
Nottingham hip fracture score, *n* (%)					**<0.001**	0.614
0–3	130	23.8	18	7.4		
4–6	350	64.1	148	61.2		
≥7	66	12.1	76	31.4		
ASA grade, *n* (%)						
1	7	1.3	0	0	**<0.001**	0.618
2	128	23.4	19	7.9		
3	312	57.1	140	57.9		
4	40	7.3	55	22.7		
5	2	0.4	3	1.2		
Not recorded	56	10.3	25	10.3		
Pre‐fracture residence, *n* (%)					**<0.001**	0.572
Home/Sheltered housing	433	79.3	129	53.3		
Care/Nursing home	92	16.8	93	38.4		
Acute hospital	21	3.8	20	8.3		
Injury location, *n* (%)					**0.001**	0.314
Outdoor	111	20.3	33	13.6		
Home/Indoor	409	74.9	180	74.4		
In hospital	25	4.6	29	12		
Missing	1	0.2	0	0		
Major comorbidity, *n* (%)						
Not present	Reference		Reference			
Cardiovascular	343	62.8	160	66.1	0.302	0.086
Renal	104	19	79	32.6	**<0.001**	0.323
Pulmonary	155	28.4	84	34.7	**0.09**	0.136
Dementia	154	28.2	105	43.4	**<0.001**	0.338
Cancer	40	7.3	36	14.9	**0.001**	0.242
Diabetes mellitus	92	16.8	51	21.1	0.187	0.108
COVID‐19 status, *n* (%)					**<0.001**	0.335
COVID‐19 negative	470	86.1	176	72.7		
COVID‐19 positive	76	13.9	66	27.3		
Timing of COVID‐positive diagnosis (%)					**<0.001**	0.357
During index stay	29	38.2	35	53.0		
After index stay	47	61.8	31	47.0		
Mean time from index admission date, days (SD)	97.7	116.3	53.7	84.3	**0.014**	0.436

*Note*: Differences with a *p*‐value < 0.1 are highlighted in bold.

Abbreviation: SMD, standardised mean difference.

^a^ Chi‐square for categorical variables, independent pairs t‐test for continuous variables.

**TABLE 2:** Patient demographics, nottingham hip fracture score, residence, length of stay, discharge destination, readmission, and mortality according to COVID‐19 status

	COVID‐19 negative	COVID‐19 positive				
Patient factor	(*n* = 646)	(*n* = 142)	*p*‐value^a^	SMD		
Mean age, yrs (SD)	79.8	10.6	83.6	8.3	**<0.001**	0.390
Sex, *n* (%)						
Male	203	31.4	57	40.1	**0.057**	0.183
Female	443	68.6	85	59.9		
Nottingham hip fracture score, *n* (%)					**<0.001**	0.399
0–3	135	20.9	13	9.2		
4–6	408	63.2	90	63.4		
≥7	103	15.9	39	27.5		
ASA grade, *n* (%)					**<0.001**	0.441
1	7	1.1	0	0		
2	134	20.7	13	9.2		
3	370	57.3	82	57.7		
4	69	10.7	26	18.3		
5	2	0.3	3	2.1		
Not recorded	64	9.9	18	12.7		
Pre‐fracture residence					**0.093**	0.197
Home/Sheltered housing	471	72.9	91	64.1		
Care/Nursing home	142	22.0	43	30.3		
Acute hospital	33	5.1	8	5.6		
Mean length of index stay, days (SD)	10.7	10.1	14.2	18.3	**0.001**	0.241
Discharge destination, *n* (%)					**<0.001**	0.818
Home/Sheltered housing	282	43.7	16	11.3		
Care/Nursing home	134	20.7	33	23.2		
Rehab/Continuing care	187	28.9	71	50.0		
Acute hospital	12	1.9	4	2.8		
Died before discharge	29	4.5	16	11.3		
Not recorded	2	0.3	2	1.4		
Readmitted after index stay						
No	461	71.4	98	69.0	0.648	0.051
Yes	185	28.6	44	31.0		
Reason for readmission, *n* (%)					**<0.001**	1.032
COVID‐19	0	0	9	6.3		
Falls (No further fracture)	20	3.1	4	2.8		
Falls (with further fracture)	23	3.6	9	6.3		
Delirium	10	1.5	2	1.4		
Functional decline	6	0.9	1	0.7		
Renal/Urinary	12	1.9	2	1.4		
Gastrointestinal	20	3.1	2	1.4		
Respiratory (non‐COVID‐19)	28	4.3	3	2.1		
Cardiac	15	2.3	1	0.7		
Cerebrovascular accident	3	0.5	0	0		
Planned surgery	4	0.6	2	1.4		
Other	40	6.2	5	3.5		
Not recorded	4	0.6	4	2.8		
No readmission	461	71.4	98	69.0		
Death within 365 days, *n* (%)					**<0.001**	0.407
No	470	72.8	76	53.5		
Yes	176	27.2	66	46.5		
Survival from injury, *d* (%)					**<0.001**	0.437
≤30	66	10.2	33	23.2		
31–60	41	6.3	14	9.9		
61–90	16	2.5	5	3.5		
91–365	53	8.2	14	9.9		
>365	470	72.8	76	53.5		
Mean survival time,^b^ days (SD)	91.8	99.2	78.0	98.9		
Principal cause of death, *n* (%)					**<0.001**	0.940
COVID‐19	0	0	40	28.2		
Renal/Urinary	2	0.3	0	0		
Gastrointestinal	8	1.2	1	0.7		
Respiratory (non‐COVID‐19)	34	5.3	10	7.0		
Cardiac	9	1.4	1	0.7		
Cerebrovascular accident	8	1.2	1	0.7		
Other	40	6.2	3	2.1		
Hip fracture	9	1.4	0	0		
Not recorded	74	11.5	13	9.2		
Not applicable	462	71.5	73	51.4		
Did COVID‐19 contribute to death? *n* (%)					**<0.001**	1.368
No	186	28.8	7	4.9		
Yes	0	0	61	43.7		
Not recorded	1	0.2	0	0		
Not applicable	459	71.1	73	51.4		

*Note*: Differences with a *p*‐value < 0.1 are highlighted in bold.

Abbreviation: SMD, standardised mean difference.

^a^ Chi‐square for categorical variables, independent pairs *t*‐test for continuous variables.

^b^ Only patients that died within 365 days of admission.

### 3.1 Prevalence of COVID‐19 and mortality at one year: Univariate analyses

**FIGURE 1:**
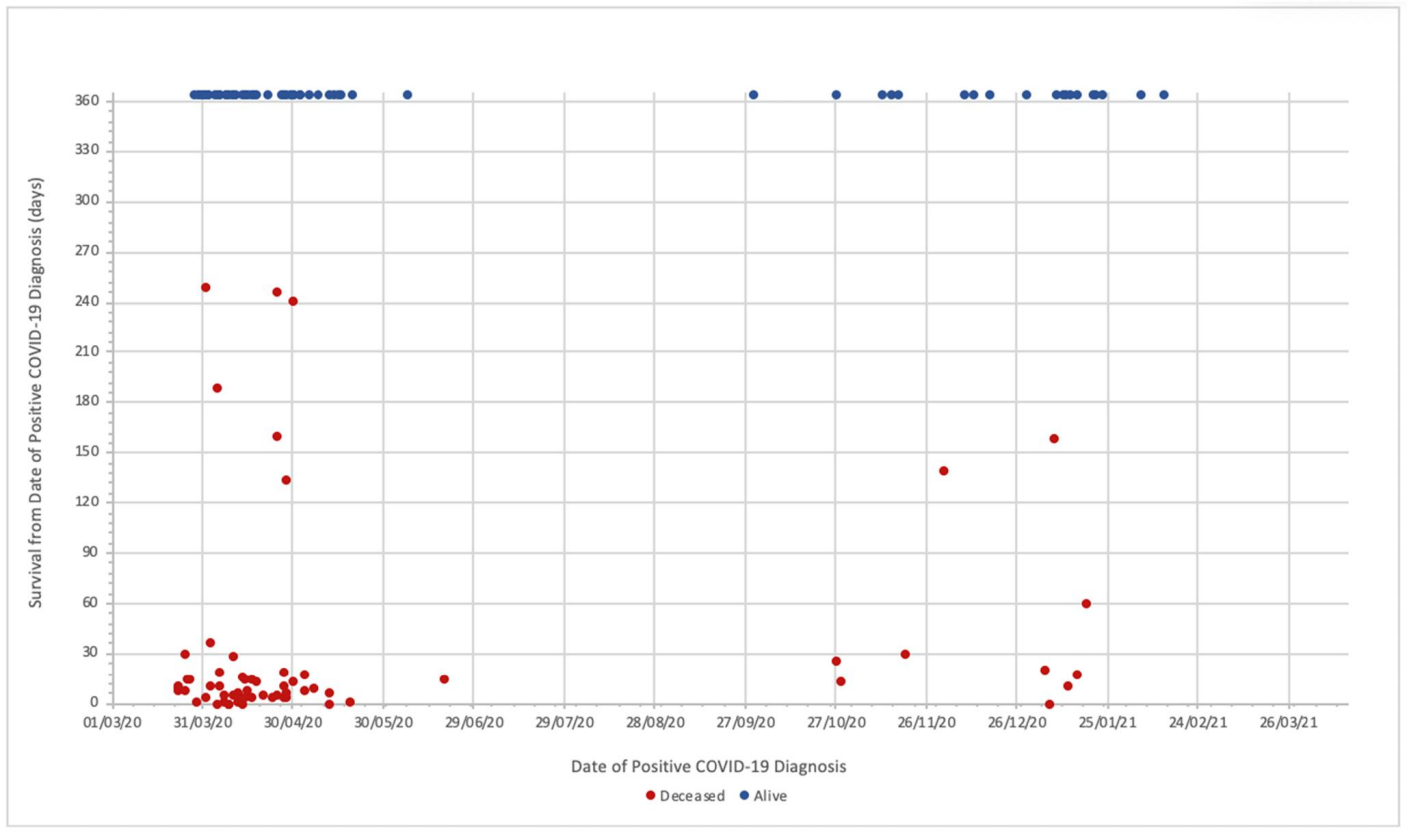
Timing of positive COVID‐19 cases and the duration of survival from date of COVID‐19 diagnosis

There were 142/788 (18.0%) patients that were diagnosed with COVID‐19 within 365 days of the date of index hip fracture admission (Table 2). There were 64/142 (45.1%) patients that were diagnosed with COVID‐19 during the index admission, and 78/142 (54.9%) that received a positive COVID‐19 diagnosis after discharge from the acute stay. Of the 78 COVID‐19 diagnoses made after the acute stay 34 (43.5%) were made within 30 days of discharge, and 25/34 (73.5%) were made in patients that had been discharged to downstream hospital settings. COVID‐19 was documented as a contributing factor in 61/242 (25.2%) of deaths and was the primary cause (as stated on the Medical Certificate of Cause of Death) in 40/242 (16.5%) patients, making it the most frequent single cause of death reported. Figure 1 demonstrates the timing of positive COVID‐19 cases and the duration of survival from the date of COVID‐19 diagnosis.

**FIGURE 2:**
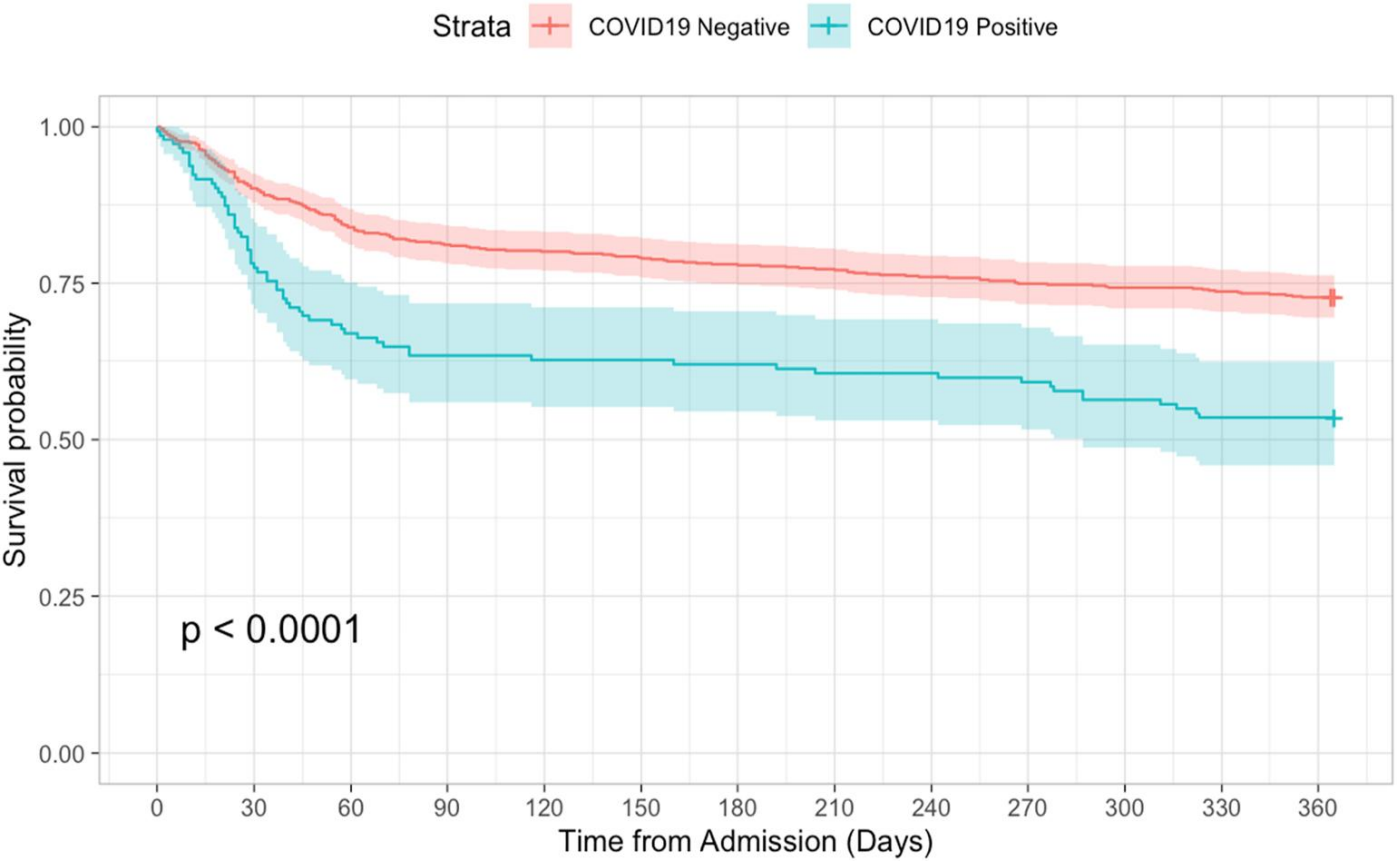
Kaplan‐Meier curve for survival at 365 days following index admission date according to COVID‐19 status (Negative = red line, Positive = blue line). Log rank *p* < 0.0001; 72.8% versus 53.5% survival at 365 days

**FIGURE 3:**
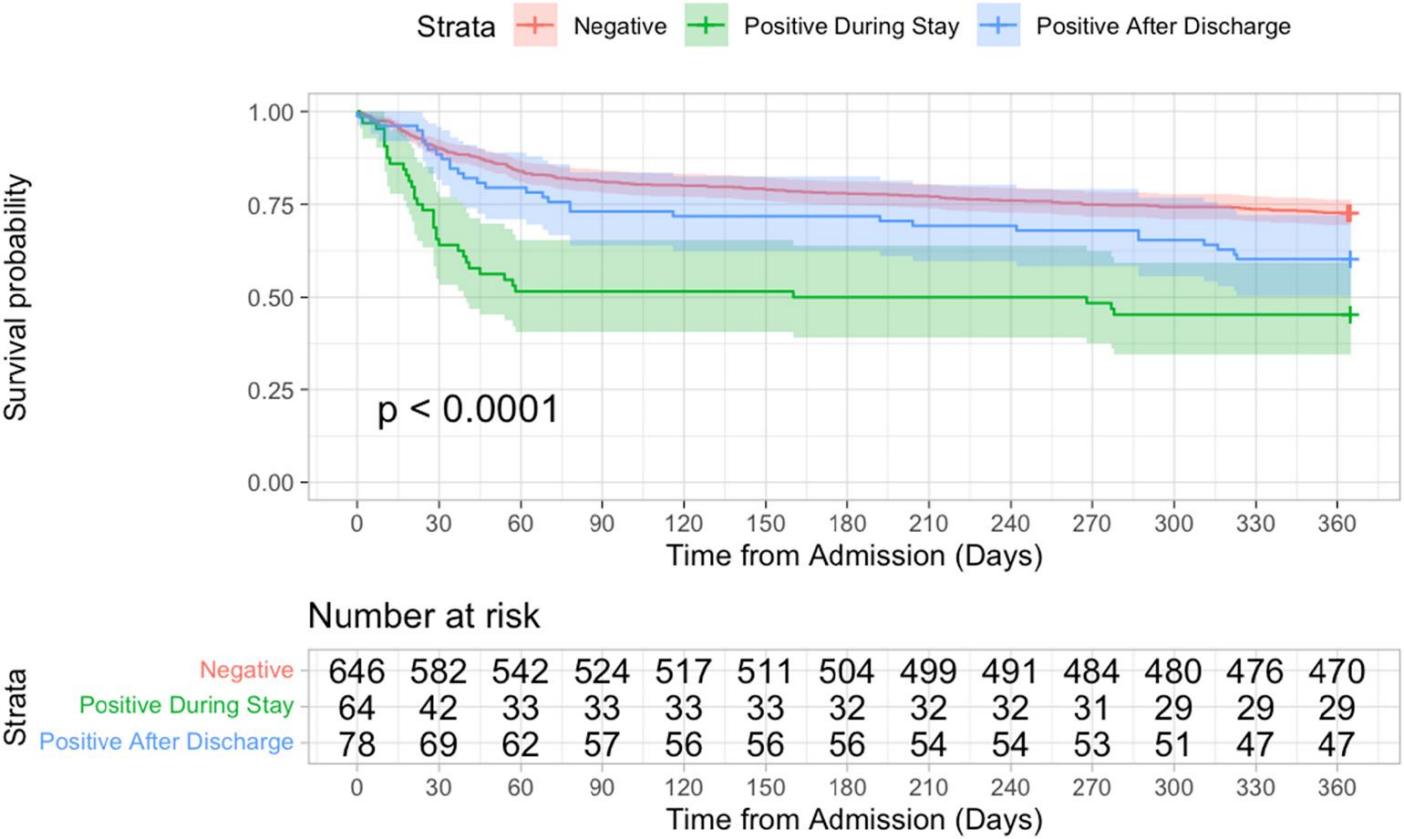
Kaplan‐Meier curve for survival at 365 days following index admission date according to COVID‐19 status. Survival at 365 days: 72.8% for COVID‐Negative (red line); 45.3% for COVID‐Positive During Index Stay (green line), and 60.3% for COVID‐Positive After Index Stay (blue line). Log rank tests demonstrated a significant (*p* < 0.05) difference between each of the groups

The 1‐year mortality rate for patients that were diagnosed with COVID‐19 at any time was significantly higher than for those that did not have COVID‐19 (46.5% vs. 27.2%, *p* < 0.001, log rank test (Figure 2)). Further, the rate of mortality at one year for patients that were diagnosed with COVID‐19 during the index hospital stay was significantly lower than for patients diagnosed with COVID‐19 after discharge (54.7% vs. 39.7%, *p* = 0.025, log rank test), and mortality rates for both groups were significantly lower than for patients that did not have COVID‐19 at any time (*p* < 0.001 and *p* = 0.025 respectively, log rank test (Figure 3). The 1‐year mortality rate for the 25 patients who were discharged to a downstream hospital setting and diagnosed with COVID‐19 within 30 days of discharge of the acute admission hospital stay was 44.0%.

### 3.2 Effect of COVID‐19 on mortality at one year: Multivariate analyses

Logistic regression analysis was used to determine factors positively associated with a positive COVID‐19 diagnosis. Factors that demonstrated an association (*p* < 0.1) with an increased likelihood of COVID‐19 on univariable analysis were included in a multivariable model. Those with a significant (*p* < 0.05) association with a COVID‐19 diagnosis on multivariable analysis included: age, male sex and higher ASA grade (Table 3). These factors were included in Cox proportional hazard regression analysis to determine the independent effect of COVID‐19 status on on‐year survival (Table 4). A COVID‐19 diagnosis at any time was independently associated with a 50% increased risk of death within a year of injury (HR 1.50, 95%CI 1.13–2.01, *p* = 0.006). A positive COVID‐19 diagnosis during the acute admission was associated with a two‐fold increased likelihood of death within 365 days of admission (HR 2.03, 95%CI 1.40–2.93, *p* < 0.001). A COVID‐19 diagnosis made after discharge from the acute hospital stay was not associated with a significantly increased risk of death at one year when controlling for confounding factors (HR 1.16, 95%CI 0.78–1.70, *p* = 0.462).

**TABLE 3:** Logistic regression analysis of factors associated with a positive COVID‐19 diagnosis

Variable	OR (univariable)	OR (multivariable)
Age		
Each increasing year	**1.04 (1.02–1.06, p < 0.001)**	**1.03 (1.00–1.06, p = 0.044)**
Sex		
Female	Reference	Reference
Male	**1.46 (1.00–2.12, p = 0.046)**	**1.69 (1.10–2.1, p = 0.018)**
ASA grade		
1	N/A	N/A
2	Reference	Reference
3	**2.28 (1.27–4.42, p = 0.009)**	1.59 (0.83–3.04, *p* = 0.164)
4	**3.88 (1.91–8.25, p < 0.001)**	**2.36 (2.09–5.11, p = 0.030)**
5	**15.46 (2.37–125.83, p = 0.004)**	**10.66 (1.51–75.54, p = 0.018)**
Nottingham hip fracture score		
0–3	Reference	Reference
4–6	**2.29 (1.28–4.41, p = 0.008)**	1.01 (0.47–2.16, *p* = 0.980)
≥7	**3.93 (2.04–8.01, p < 0.001)**	1.20 (0.40–3.59, *p* = 0.747)
Pre‐fracture residence		
Home/Sheltered housing	Reference	Reference
Care/Nursing home	**1.57 (1.04–2.35, p = 0.031)**	0.83 (0.46–1.52, *p* = 0.550)
Acute hospital	1.35 (0.52–2.68, *p* = 0.068)	0.38 (0.20–2.38, *p* = 0.141)
Injury location		
Outdoor	Reference	Reference
Indoor	0.72 (0.38–1.45, *p* = 0.336)	0.52 (0.19–1.42, *p* = 0.202)
In hospital	0.48 (0.22–1.07, *p* = 0.068)	0.47 (0.15–1.44, *p* = 0.185)
Major comorbidity		
Cardiovascular disease	1.20 (0.87–1.65, *p* = 0.266)	N/A
Renal disease	**1.76 (1.18–2.62, p = 0.005)**	1.19 (0.77–1.85, *p* = 0.438)
Pulmonary disease	1.08 (0.73–1.59, *p* = 0.697)	N/A
Dementia	**1.74 (1.20–2.52, p = 0.003)**	2.55 (0.90–2.65, *p* = 0.111)
Cancer	**0.67 (0.31–1.27, p = 0.029)**	0.55 (0.26–1.17, *p* = 0.119)
Diabetes	1.32 (0.90–1.92, *p* = 0.157)	N/A

*Note*: Differences with a *p*‐value < 0.1 are highlighted in bold.

**TABLE 4:** Cox proportional hazard regression analysis of the independent effect of a positive COVID‐19 diagnosis on 365‐day mortality, adjusted for factors associated with a positive COVID‐19 diagnosis

Covariate	HR (multivariable)
Age	
Each increasing year	**1.04 (1.03–1.06, p < 0.001)**
Sex	
Female	Reference
Male	**1.96 (1.51–2.54, p < 0.001)**
ASA grade	
1	N/A
2	Reference
3	1.72 (1.12–2.65, *p* = 0.013)
4	**3.91 (2.41–6.32, p < 0.001)**
5	**3.88 (1.15–13.14, p = 0.029)**
COVID‐19 status	
Negative	Reference
Positive (any time)	**1.50 (1.13–2.01, p = 0.006)**
Positive (during index stay)	**2.03 (1.40–2.93, p < 0.001)**
Positive (after index stay)	1.16 (0.78–1.70, *p* = 0.462)

*Note*: Concordance = 0.70 (SE = 0.016). Differences with a *p*‐value < 0.1 are highlighted in bold.

### 3.3 Readmissions and associated morbidity

There were 221/788 (28.0%) patients that were readmitted to an acute hospital within 365 days of presentation with hip fracture; 56/788 (7.1%) patients were admitted for falls (making falls the most common reason for readmission) and in 32 cases a new fracture had been sustained (Table 2). Non‐COVID respiratory system conditions were also common reasons for readmission (31/788; 3.9%), and in 9/788 (1.1%) cases COVID‐19 was given as the reason for readmission.

### 3.4 The effect of timing of a post‐discharge COVID‐19 diagnosis on survival

A sub‐group analysis of 1‐year mortality following fracture was conducted for the 78 patients that were COVID‐positive after discharge from the acute stay. These patients were dichotomised into those diagnosed with COVID‐19 before or after 1^st^ January 2021. This date was selected because it was assumed that the majority of hip fracture patients in Scotland are likely to have received COVID‐19 vaccination by this time and might reasonably be expected to demonstrate the protective effects conferred by the vaccine observed in patients of similar characteristics (Sheikh et al., 2021). There were 61/78 (78.2%) patients diagnosed COVID‐positive before 1^st^ January 2021, and 1‐year mortality was 41.0%. There were 16/78 (20.5%) patients diagnosed COVID‐positive after 1^st^ January 202, and in this group the mortality 1 year after fracture was 31.2%.

### 3.5 Timing of deaths among patients with COVID‐19

The median survival time from initial presentation for COVID‐negative patients was 90.2 days compared to 34.0 days for COVID‐positive patients. The median time from presentation to COVID‐19 diagnosis was 17.0 days (for those diagnosed during the index admission) and 49.0 days (for those diagnosed following discharge). The median time from COVID‐19 diagnosis to death was 11.0 days for all COVID‐positive patients (10.0 and 11.0 days for those diagnosed during the index admission and after discharge, respectively). Only 10/66 (15.2%) deaths among COVID‐positive patients occurred beyond 30 days of COVID‐19 diagnosis, which is demonstrated by clustering of these deaths shortly following diagnosis (Figure 1).

### 3.6 Missing data

Missingness analysis revealed that American Society of Anaesthesiologists (ASA) grade was missing for 81/788 (10.2%) of patients, and of these 61 (75.3%) related to patients from two of the study centres. Further investigation determined that this is accounted for by data collection omission relating to the unavailability of records, rather than to the respective subjects, therefore these data were handled as missing completely at random (MCAR).

## 4 DISCUSSION

This nationwide multicentre cohort study provides the first long‐term follow‐up of patients that suffered a hip fracture during the COVID‐19 pandemic. It has demonstrated that COVID‐19 affecting these patients was independently associated with a 50% increased risk of death within a year of fracture. COVID‐19 was a contributing factor in at least a quarter of all observed deaths during the study period. The majority of deaths in COVID‐positive patients occurred within a month of contracting the disease, and patients diagnosed with COVID‐19 during the acute hip fracture admission were especially vulnerable, with a two‐fold increased risk of death within a year of injury compared to those without COVID‐19. Nearly half of the COVID‐19 diagnoses made after the acute stay occurred within a month of discharge, and the majority of these were in patients in continuing care institutions.

Although the 1‐year mortality rate of patients that were diagnosed with COVID‐19 within a year of hip fracture was significantly higher than that of patients without COVID‐19 (46.5% vs. 27.2% respectively), when controlling for confounding factors COVID‐19 only had a significant effect on mortality when COVID‐19 arose during the index hip fracture admission. This may be due to a ‘double‐hit’ effect in which SARS‐CoV‐2 infection contracted during a period of particular vulnerability (fracture and surgery) is especially deleterious to health. This vulnerability to contracting, and dying from, COVID‐19 appears to be a feature of fragility fracture patients but is not demonstrated in orthopaedic patients undergoing planned essential surgery (Clement et al., 2021; Clement, Hall, et al., 2020).

The 30‐day mortality rate for COVID‐negative patients was 10.2% in the current study, which is higher than the rate observed in pre‐COVID years (typically <7%), and higher than the overall 30‐day mortality rate observed by the SHFA for all patients (COVID‐positive and negative) in 2020 (Public Health Scotland, 2021). This may reflect natural variation in patient survival since the current study only presents data for patients admitted over a short period, or may indicate that cases of COVID‐19 went undetected. Another possible explanation is that mortality was higher for all patients due to the major disruptions experienced by hip fracture services, including the breakdown of multidisciplinary hip fracture services, reduced access to operating theatres, and management of patients on disparate non‐specialist wards that is associated with reduced quality of care and increased risk of nosocomial infection (Hall, Clement, MacLullich, Ojeda‐Thies, et al., 2021; McMurdo & Witham, 2013; Patel et al., 2020). Acute hip fracture services require the delivery of highly specialised multidisciplinary care to meet the complex needs of patients, and adherence to evidence‐based national standards has been shown to be associated with better outcomes, including lower mortality rates, shorter length of hospital stay, and a higher chance of discharge back to the pre‐fracture level of care (Farrow et al., 2018, 2020, 2021; Metcalfe et al., 2019).

Almost half of the cases of COVID‐19 diagnosed within a year of a hip fracture were identified within the first 30 days of discharge from the acute stay, and the majority of these cases were in patients who were discharged to a downstream care facility (i.e. an inpatient or residential unit other than an acute hospital). This is a concern given the high incidence of nosocomial infection demonstrated by our previous study, which showed that around half of all COVID‐19 cases diagnosed early in the patient journey (within 30 days of initial presentation) were presumed to be hospital‐acquired (Hall, Clement, MacLullich, White, et al., 2021). The findings of the current study, which demonstrate a large proportion of cases diagnosed after discharge to continuing care settings, indicates that the rate of nosocomial transmission remained high within downstream care facilities. The 1‐year mortality rate of 44% observed for these patients further supports the introduction of robust infection prevention and control measures in healthcare settings, pathways to protect patients that have previously been identified as especially vulnerable, and the development of reliable intra‐ and inter‐unit contract tracing. These measures remain important despite the widespread uptake of COVID‐19 vaccination in this at‐risk population because of the emergence of vaccine resistant coronavirus strains. Interestingly, the mortality rate of patients diagnosed with COVID‐19 after discharge was slightly lower for cases identified after 1^st^ January 2021. This may reflect a survival advantage conferred by vaccination, but further work is required before a causal link can be drawn in the hip fracture population.

The majority of deaths in patients with a history of COVID‐19 occurred within a month of COVID‐19 diagnosis, after which the risk of death at each time point for COVID‐positive patients was similar to COVID‐negative patients. This may indicate that the increased mortality risk conferred by COVID‐19 is particularly relevant during the acute infection, or during a period of increased vulnerability due to injury or surgery. Although deaths occurring later in this group were less common, COVID‐19 may still have an enduring deleterious effect on health and level of function following hip fracture. Prior to sustaining a hip fracture over 70% of patients in the current study lived in a low‐care needs setting (home or sheltered housing), but only 38% were discharged from the acute inpatient stay to an equivalent level of care and a large proportion required continuing care in a higher‐care needs facility. A significantly greater proportion of COVID‐negative patients were discharged back to low‐care demand settings compared to COVID‐positive patients, which may indicate that patients who are affected by COVID‐19 and survive beyond the acute stay may have increased frailty and higher care needs. More work is required to better determine the long‐term effects of COVID‐19 on frailty, performance status, and health and social care needs.

This study has several strengths. Firstly, the data were collected using a bespoke audit system by specialist local audit coordinators and clinicians who were familiar with each study centre and their records processes. Secondly, the data collected in the follow‐up study were cross‐referenced with the original datasets in order to verify their accuracy and assess for completeness. Thirdly, the study was carried out at a national level and represents the care of hip fracture patients across Scotland. This study provides important follow‐up data related to longer‐term outcomes in hip fracture patients during the COVID‐19 pandemic and, as evidence emerges from other clinical audits, the findings could be combined to inform clinicians and health and social care administrators about the ongoing needs of this vulnerable patient group (Hall, Duckworth, et al., 2021). Furthermore, the findings of this large nationwide study of hip fracture patients may be generalisable to other frail populations, and are thus relevant to the ongoing strategic and clinical management of the COVID‐19 pandemic and future communicable disease outbreaks.

Some limitations to the study should be acknowledged. The prevalence of COVID‐19 is likely to have been underestimated due to the lack of widespread testing capacity early in the pandemic, and because of the frequency of ‘atypical’ features of COVID‐19 in the older population (BGS, 2020). The unavailability of a death certificate in one‐third of patients, which was typically due to a death occurring in the community rather than an acute hospital may have also resulted in the underestimation of the number of deceased patients that were judged to have died from COVID‐19. Additionally, the data presented are from patients that sustained a hip fracture early in the COVID‐19 pandemic and prior to the availability of vaccination, which has been shown to reduce incidence and severity of COVID‐19 (Sheikh et al., 2021). This may be considered a strength in light of recent concerns about incomplete vaccination and vaccination booster programmes, as well as the emergency of SARS‐CoV‐2 strains that demonstrate resistance to both vaccine‐mediated and previous infection‐mediated immunity (Wang et al., 2021). Similar such studies are unlikely to be feasible now. Future work should utilise existing clinical audit mechanisms to monitor long‐term effects on a population‐wide level, and the compilation of data from multiple national hip fracture audit programmes (adhering to a minimum common clinical dataset) would ensure generalisability to a range of regions and healthcare contexts (Hall, Duckworth, et al., 2021; Johansen et al., 2022).

## 5 CONCLUSION

Almost half of the hip fracture patients affected by COVID‐19 had died within a year of injury and COVID‐19 was a contributing factor in a quarter all observed deaths. A positive COVID‐19 status at any point within a year of hip fracture was independently associated with a 50% increased risk of death in the same period, and the majority of deaths in COVID‐positive patients occurred within a month of diagnosis of the disease. When adjusting for confounding factors, only a COVID‐19 diagnosis made during the acute admission was associated with an increased mortality risk, and this group had a two‐fold increased risk of death within a year of fracture compared to COVID‐negative patients. This may reflect a ‘double hit’ insult in which patients are more vulnerable to contracting and dying from COVID‐19 during a period of acute illness and emergency surgery.

## AUTHOR CONTRIBUTIONS

Hall: Conceptualisation, Methodology, Formal analysis, Investigation, Data curation, Writing ‐ original draft, Project administration; Clement: Conceptualisation, Methodology, Investigation, Writing ‐ original draft, Supervision; IMPACT Restart Group (list of co‐authors’ names provided in submission): Investigation; MacLullich: Writing ‐ review and editing, Supervision; White: Writing ‐ review and editing, Supervision; Duckworth: Conceptualisation, Methodology, Investigation, Writing ‐ original draft, Supervision.

## CONFLICT OF INTEREST

All authors declare that they have no conflict of interest or financial/non‐financial interests related to this work.

## ETHICS STATEMENT

Data were collected by clinicians or specialist hip fracture audit coordinators local to each unit, and anonymised data were submitted to the central IMPACT Project Lead Team. All data were handled in accordance with UK Caldicott principles. The study was designation as a clinical audit and was conducted with the oversight of the Scottish Hip Fracture Audit Research Group.

## Data Availability

The data that support the findings of this study are available on request from the corresponding author. The data are not publicly available due to privacy or ethical restrictions.

## IMPACT‐Revisited Group

The following individuals contributed significantly to this work as members of the IMPACT‐Revisited Group and satisfy the ICJME criteria for recognition as co‐authors.

**Table:**

Ablett	Andrew	Harding	Thomas
Abu‐Rajab	Rashid	Howieson	Alan
Aithie	Joanna MS	Jenkins	Paul J
Bashyam	Jeswant	Kennedy	Matthew
Bell	Jean	McKay	Pamela
Bell	Stuart	Mighton	Shuna
Brennan	Joseph	Murphy	Luke
Brunt	Andrew	Murray	Alastair W
Burns	Shirley	Owens	AnneMarie
Carnegie	Carol	Rae	Fraser J
Clarke	Jon V	Sharma	Sidharth
Collins	Grace	Simpson	James
Dall	Graham F	Stewart	Claire
Davison	Martin	Weerasuriya	Thisara
Farrow	Luke	Wood	Janet
Fraser	Ewen	Christopher W Gee	
